# Role of VEGF-A in angiogenesis promoted by umbilical cord-derived mesenchymal stromal/stem cells: in vitro study

**DOI:** 10.1186/s13287-016-0305-4

**Published:** 2016-03-22

**Authors:** Irina Arutyunyan, Timur Fatkhudinov, Evgeniya Kananykhina, Natalia Usman, Andrey Elchaninov, Andrey Makarov, Galina Bolshakova, Dmitry Goldshtein, Gennady Sukhikh

**Affiliations:** Research Center for Obstetrics, Gynecology and Perinatology of Ministry of Healthcare of the Russian Federation, 4 Oparina Street, Moscow, 117997 Russia; Scientific Research Institute of Human Morphology, 3 Tsurupa Street, Moscow, 117418 Russia; Pirogov Russian National Research Medical University, Ministry of Healthcare of the Russian Federation, 1 Ostrovitianov Street, Moscow, 117997 Russia; Laboratory of Regenerative Medicine, Research Center for Obstetrics, Gynecology and Perinatology, 4 Oparin Street, Moscow, 117997 Russia; Research Center of Medical Genetics, 1 Moskvorechie Street, Moscow, 115478 Russia

**Keywords:** Mesenchymal stromal cells, Multipotent, Wharton jelly, Endothelial cells, In vitro techniques, Vascular endothelial growth factor-A, Angiogenesis inducing agents, CD31 antigen, Cell migration assays, Extracellular matrix

## Abstract

**Background:**

Mesenchymal stromal/stem cells derived from human umbilical cord (UC-MSCs) uniquely combine properties of embryonic and postnatal MSCs and may be the most acceptable, safe, and effective source for allogeneic cell therapy e.g. for therapeutic angiogenesis. In this report we describe pro-angiogenic properties of UC-MSCs as manifested *in vitro*.

**Methods:**

UC-MSCs were isolated from human Wharton’s jelly by enzymatic digestion. Presence of soluble forms of VEGF-A in UC-MSC-conditioned media was measured by ELISA. Effects of the conditioned media on human umbilical vein-derived endothelial EA.hy926 cells proliferation were measured by MTT-assay; changes in cell motility and directed migration were assessed by scratch wound healing and transwell chamber migration assays. Angiogenesis was modeled *in vitro* as tube formation on basement membrane matrix. Progressive differentiation of MSCs to endothelioid progeny was assessed by CD31 immunostaining.

**Results:**

Although no detectable quantities of soluble VEGF-A were produced by UC-MSCs, the culture medium, conditioned by the UC-MSCs, effectively stimulated proliferation, motility, and directed migration of EA.hy926 cells. In 2D culture, UC-MSCs were able to acquire CD31^+^ endothelial cell-like phenotype when stimulated by EA.hy926-conditioned media supplemented with VEGF-A165. UC-MSCs were capable of forming unstable 2D tubular networks either by themselves or in combinations with EA.hy926 cells. Active spontaneous sprouting from cell clusters, resulting from disassembling of such networks, was observed only in the mixed cultures, not in pure UC-MSC cultures. In 3D mode of sprouting experimentation, structural support of newly formed capillary-like structures was provided by UC-MSCs that acquired the CD31^+^ phenotype in the absence of exogenous VEGF-A.

**Conclusion:**

These data suggest that a VEGF-A-independent paracrine mechanism and at least partially VEGF-A-independent differentiation mechanism are involved in the pro-angiogenic activity of UC-MSCs.

## Background

The concept of therapeutic angiogenesis stems from understanding the importance of adequate microvascular supply for growth and regeneration of affected tissues; it refers to actions performed to facilitate revascularization of ischemic tissues. As long as the direct delivery of exogenous cytokines and growth factors is ineffective, primarily because of their rapid elimination in vivo [1], expert opinions agree that the most promising approach for therapeutic angiogenesis is represented by stem cell therapy using multipotent mesenchymal stromal/stem cells (MSCs) because it comprises simultaneous activation of multiple mechanisms (paracrine, replacement, trophic, immunomodulatory) to provide support on different stages of formation and maturation of blood vessels [2–4]. Most of the research in this field is performed on bone marrow-derived MSCs (which represent a ‘gold standard’) or adipose tissue-derived MSCs; both lineages have certain angiogenic potential and implement it in a similar manner [2, 5]. The field of therapeutic angiogenesis is currently expanded by using MSCs from other sources, importantly from the umbilical cord and placenta. Perinatal stem cells share characteristics with both embryonic and adult stem cells because they may exhibit pluripotency as well as multipotent tissue maintenance, thus representing a bridge between embryonic and adult stem cells [6]. Umbilical cord-derived MSCs (UC-MSCs) have distinct biological properties: they are highly proliferative and enriched in transcriptionally active genes related to liver or cardiovascular system development and function. Besides, UC-MSCs exhibit superior grades of plasticity and immunomodulatory activity, lack tumorigenicity, and are considered the best resource for allogeneic transplantation [7–10].

Although therapeutic efficacy of UC-MSC transplantation to ischemic tissue is demonstrated in vivo [11], the understanding of how these cells implement their pro-angiogenic potential is far from complete, and there is a certain controversy among reports on this subject. The inner space of umbilical cord, net of large vessels, is occupied by special connective tissue, the Wharton jelly, which is very loose and rich in gel-like ground substance. Complete absence of microcirculatory vessels from Wharton jelly may indicate some anti-angiogenic properties of this microenvironment. The assumption is partially supported by recent in vitro studies. For instance, Kuchroo et al. [12] show that UC-MSCs do not produce detectable amounts of soluble vascular endothelial growth factor (VEGF)-A protein (strictly speaking, they probably do because the corresponding gene is actively transcribed, but apparently this secreted VEGF-A is saturated by soluble VEGF-A-specific receptors that are also secreted by UC-MSC and act as a buffer); at the same time, the authors observe certain stimulating influences of UC-MSCs on umbilical vein-derived endothelial cells in vitro and suggest the existence of some alternative, VEGF-independent mechanism for this stimulation. A comparative study by Amable et al. [13] describe a shifted balance of pro-angiogenic and anti-angiogenic factors in UC-MSC secretome, as compared with MSCs from other conventional sources. Ways in which UC-MSCs interact with endothelial cells and their own responses to inducers of endothelial differentiation represent an open issue. In the current study, the pro-angiogenic activity of human UC-MSCs is challenged in vitro by modeling of angiogenesis using 2D and 3D artificial matrices. Several specifically addressed problems include the importance of VEGF-A, modes of UC-MSC cooperation with umbilical vein-derived endothelial EA.hy926 cells, and UC-MSC ability to acquire CD31^+^ phenotypes under various stimulations.

## Methods

### Cell cultures

The study involving human material was approved by the Ethics Committee at the Research Center for Obstetrics, Gynecology and Perinatology. Written informed consent was obtained from all participants prior to the study.

MSCs were isolated from human umbilical cords (*n* = 5). The material was rinsed in phosphate-buffered saline (PBS) with 1 mg/ml cefazolin (Sintez, Kurgan, Russia) and cut into 3–4 cm pieces. After removal of blood vessels and amnion, the Wharton jelly was chopped into smaller fragments with scissors. The fragments were incubated with 200 U/ml collagenase type I (PanEco, Moscow, Russia) and 40 U/ml dispase (Invitrogen, Waltham, MA, USA) for 60 minutes at 37 °C. After the addition of fetal bovine serum (FBS; GE Healthcare, Pittsburg, PA, USA), the digested mixture was centrifuged at 1000 × *g* for 10 minutes at room temperature. Finally, the digested pieces were washed with serum-free Dulbecco’s modified Eagle’s medium (DMEM; PanEco) and cultured in growth medium (DMEM/F12 supplemented with 10 % FBS and 1 % penicillin–streptomycin (PanEco)) in a humidified incubator at 37 °C under a 5 % CO_2_ atmosphere.

UC-MSCs were characterized according to the minimal criteria to define human MSCs as proposed by the Mesenchymal and Tissue Stem Cell Committee of the International Society for Cellular Therapy [14]. For immunophenotype analysis, cells were labeled for 30 minutes at room temperature using the BD Stemflow™ hMSC Analysis Kit (BD Biosciences, Pharmingen, San Diego, CA, USA). After being fixed with 4 % paraformaldehyde (SERVA Electrophoresis, Heidelberg, Germany), the cells were analyzed on a FACScalibur using CellQuest software (BD Biosciences). The StemPro® Adipogenesis Differentiation Kit, the StemPro® Osteogenesis Differentiation Kit, and the StemPro® Chondrogenesis Differentiation Kit (Gibco, Life Technologies, Carlsbad, CA, USA) were used to demonstrate the differentiation capacity of UC-MSCs in accordance with the manufacturer’s instructions.

Human endothelial EA.hy926 cells were derived from the American Type Culture Collection (Manassas, VA, USA). Established in 1983 by fusing primary human umbilical vein endothelial cells (HUVEC) with a thioguanine-resistant clone of the human lung adenocarcinoma cell line A549/8, EA.hy926 cells represent a widely-used endothelial cell line expressing endothelin-1, Weibel-Palade bodies, prostacyclin, factor VIII-related antigen, and endothelial adhesion molecules ICAM-1 and VCAM-1 [15]. This line was chosen for its highly specific functions that are characteristic of the human vascular endothelium combined with advantages of immortality, stability through passage number, and high reproducibility of the properties [16, 17].

### Immunofluorescence

Cells were fixed with 4 % paraformaldehyde (SERVA Electrophoresis) for 10 minutes at room temperature. After two washes with PBS, the cells were blocked for 5 minutes with Protein Block (Abcam, Cambridge, MA, USA) at room temperature and then incubated overnight at 4 °C with antibodies against CD31 (ab24590; Abcam). After washing with PBS, the cells were incubated with fluorescein isothiocyanate (FITC)-conjugated antimouse IgG (ab6810; Abcam) for 1 hour in the dark. Cell nuclei were stained with 4′,6-diamidino-2-phenylindole (DAPI; Sigma-Aldrich, St. Louis, MO, USA). The cells were observed under the Leica DM 4000 B fluorescent microscope (Leica Microsystems, Heidelberg, Germany).

### Preparation of conditioned media

At 100 % confluence, the cells (UC-MSCs or EA.hy926) were washed with serum-free DMEM, and the media were replaced with fresh growth media. After 24, 48, or 72 hours, the media were collected and centrifuged at 2800 × *g* for 5 minutes, filtered through a 0.22 μm filter (GE Osmonics Labstore, Minnetonka, MN, USA), and were then stored at –70 °C until VEGF-A quantification. The media conditioned by UC-MSCs or EA.hy926 cells for 72 hours were used in subsequent experiments.

### VEGF-A quantification

Media conditioned by EA.hy926 cells or UC-MSCs were collected after 24, 48, or 72 hours. VEGF-А-121 and VEGF-A-165 were quantified using a commercial enzyme-linked immunosorbent assay kit (#8784; Vector-Best, Novosibirsk, Russia) in accordance with the instructions of the manufacturer. Data analysis was performed using the online application (http://elisaanalysis.com/app).

### Endothelial cell proliferation assay

EA.hy926 cells were seeded in a 96-well plate (3 × 10^3^ cells in 200 μl of growth media per well). After 1, 2, or 3 days the media were replaced with UC-MSC-conditioned media, UC-MSC-conditioned media supplemented with 200 ng/ml anti-VEGF antibody (ab9570; Abcam), or fresh growth media (control wells). At day 4 the cell proliferation was measured by 3-(4,5-dimethylthiazol-2-yl)-2,5-diphenyltetrazolium bromide (MTT) assay. MTT (Sigma-Aldrich) stock solution was added to each well (to a final MTT concentration of 1.5 mg/ml). The plate was returned to a cell culture incubator for 2 hours. When the purple precipitate was clearly visible under the microscope, 100 μl of dimethyl sulfoxide (DMSO; Sigma-Aldrich) were added. After 15 minutes, the absorbance in each well was measured at 570 nm in a Multiskan GO microplate spectrophotometer (Thermo Fisher Scientific, Waltham, MA, USA). The reference wavelength was 650 nm.

### Endothelial cell transwell migration assay

The migration of EA.hy926 cells to UC-MSC-released chemoattractants was measured by transwell chamber migration assay. UC-MSCs were seeded in a 24-well plate (10^5^ cells in 600 μl of growth media per well). One-half of the wells with the cells contained anti-VEGF antibody (ab9570; Abcam) in 200 ng/ml final concentration. The same volume of growth media without cells was added to control wells. After 24 hours, inserts with a polycarbonate membrane (pore size of 8 μm) (#35224; SPL Life Sciences, Pochun, South Korea) were installed in the plate. EA.hy926 cells were seeded in the upper chambers (10^5^ cells in 250 μl of growth media). After 24, 48, or 72 hours, nonmigrating cells in the upper chamber were attentively removed with cotton swabs, and cells on the lower surface of the membrane were fixed with 4 % paraformaldehyde (SERVA Electrophoresis) and stained with DAPI (Sigma-Aldrich). The total numbers of migrated cells were then counted in eight randomly selected fields for each insert (magnification × 100) using LAS AF v.3.1.0 build 8587 (Leica Microsystems).

### Endothelial cell scratch healing assay

EA.hy926 cells were seeded in a 96-well plate (3 × 10^4^ cells in 100 μl of growth media per well). After 24 hours, each confluent cell monolayer was scratched with a WoundMaker™ tool (Essen Bioscience, Ann Arbor, MI, USA), which created 96 homogeneous scratch wounds without cell irritation. After washing with PBS, 100 μl of fresh growth media, UC-MSC-conditioned media, or UC-MSC-conditioned media supplemented with anti-VEGF antibody (ab9570; Abcam) in 200 ng/ml final concentration were added to the wells. A 36-hour time-lapse movie was created by IncuCyte ZOOM® Live-Cell Imaging Platform (Essen BioScience). Wound confluence was measured using an automated Cell Migration software module (Essen BioScience).

### In vitro tube formation assay

In this experiment we used BD Matrigel™ Basement Membrane Matrix Phenol Red Free (#356237; BD Biosciences). This matrix is highly enriched in laminin-1, collagen IV, heparan sulfate, proteoglycan, entactin/nidogen, and various growth factors. Although it does not contain all of the signature components of an endothelial basement membrane, Matrigel promotes tube formation in vitro for all endothelial cells tested to date [18].

Prior to the experiment, UC-MSCs were labeled with PKH26 (yellow–orange fluorescent dye) and EA.hy926 cells were labeled with PKH67 (green fluorescent dye) (Sigma-Aldrich) according to the manufacturer’s instructions. A total of 150 μl of chilled Matrigel was added to a 48-well plate and incubated at 37 °C for 30 minutes. UC-MSCs, or EA.hy926 cells, or 1:1 mixed UC-MSC-EA.hy926 cells (total 35 × 10^3^ cells per well) were suspended in 500 μl of growth media and were added to the solidified Matrigel. Additionally, EA.hy926 cells suspended in 500 μl of UC-MSC-conditioned media or UC-MSC-conditioned media supplemented with 200 ng/ml of anti-VEGF antibody (ab9570; Abcam) were used. After incubation on Matrigel at 37 °C in a 5 % CO_2_ chamber, morphological changes were observed under an Axiovert 40 CFL inverted microscope (Carl Zeiss, Jena, Germany). Six representative fields for each well were photographed. Images were analyzed using AxioVs40 4.8.2.0 (Zeiss, Oberkochen, Germany) to determine the length of the tubes and the number of branch points (magnification × 50).

### Endothelial differentiation of UC-MSCs in monolayer

After UC-MSCs formed the confluent monolayer, the growth media were replaced with the induction media. Three endothelial induction media were used for UC-MSC culture: EA.hy926-conditioned media mixed 1:1 with growth media; EA.hy926-conditioned media mixed 1:1 with growth media supplemented with VEGF-A-165 (#583702; BioLegend, San Diego, CA, USA), 50 ng/ml; and growth media supplemented with VEGF-A-165. Contents of FCS in the control and differentiation media were reduced to 5 % to avoid excessive cell growth. The media were replaced twice a week. At day 21, UC-MSCs were fixed with 4 % paraformaldehyde (SERVA Electrophoresis) and stained with CD31 antibodies as already described.

### Endothelial differentiation of UC-MSCs in Matrigel

Whole mount immunostaining of the 3D structure, formed by the cells in Matrigel, proved impossible due to nonspecific absorption of the antibodies by the matrix. For this reason, the analysis was performed on cryosections of secondary sprouting networks. Structures formed in Matrigel were embedded in Tissue-Tek® OCT Compound (Sakura Finetek, Torrance, CA, USA) and cut into 5–7 μm sections using a cryostat. The sections were stained with CD31 antibodies as already described.

### Statistical analysis

Data are expressed as mean ± standard deviation (SD). Student’s *t* test was used for pairwise comparisons between groups of normally distributed values, whereas the Mann–Whitney test was applied for distributions differing from normal. Multiple comparisons were done by either one-way analysis of variance (ANOVA) or ANOVA on ranks (for the cases of unconfirmed normality); *p* <0.05 was considered statistically significant.

## Results

### Characterization of cell cultures

**Fig. 1:**
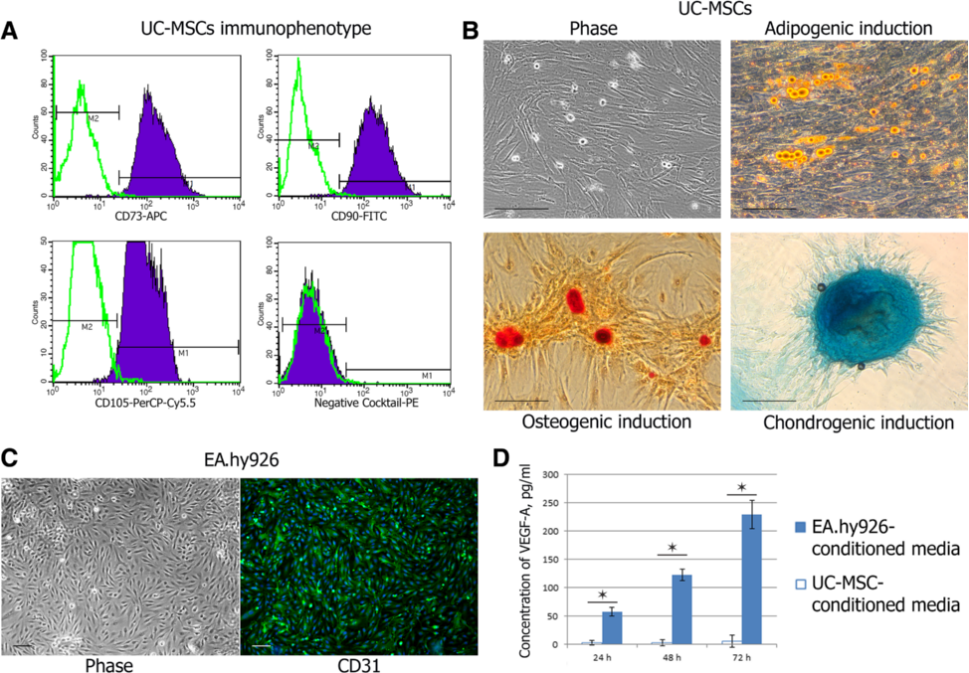
Cell culture characterization. **a** Human UC-MSC immunophenotype, positive for CD73, CD90, and CD105 and negative for CD45, CD34, CD11b, CD19, and HLA-DR. **b** Multilineage differentiation of UC-MSCs. Differentiation into adipocytes was revealed by Sudan III staining for intracellular accumulated lipids. Differentiation into osteocytes was revealed by Alizarin Red S staining for calcium mineralization. Chondrogenic differentiation was revealed by Alcian blue staining for mucopolysaccharides. Scale bar 100 μm. **c** Positive staining of EA.hy926 cells for CD31 as endothelial marker. Scale bar 100 μm. **d** Concentration of soluble forms of VEGF-A (VEGF-A-121 and VEGF-A-165) in the EA.hy926-conditioned media and the UC-MSC-conditioned media. Values are expressed as average ± SD of three replicates. *p <0.05. *h* hours, *UC-MSC* umbilical cord-derived mesenchymal stromal/stem cell, *VEGF* vascular endothelial growth factor

The cells isolated from Wharton jelly of the human umbilical cord were plastic adherent with a spindle-shaped, fibroblast-like morphology (Fig. 1b). Flow cytometry analysis showed that these cells were positive for the MSC markers CD105, CD73, and CD90 and were negative for CD11b, CD19, CD45, CD34, and HLA-DR (Fig. 1a).

UC-MSCs demonstrated multipotent differentiation potential. Sudan III staining of neutral lipid vacuoles showed that UC-MSCs could differentiate into adipocytes. Alizarin Red S staining of calcium compound crystals showed that UC-MSCs could differentiate into osteoblasts. Positive staining of mucopolysaccharides by Alcian blue indicated that UC-MSCs could differentiate into chondrocytes (Fig. 1b).

EA.hy926 cells formed a monolayer of closely apposed small polygonal cells. EA.hy926 cells were found to be positive for CD31 as endothelial marker (Fig. 1c).

### VEGF-A-121 and VEGF-A-165 quantification in the conditioned media

Within 3 days, the concentration of soluble forms of VEGF-A (VEGF-A-121 and VEGF-A-165) in EA.hy926-conditioned media gradually increased from 57.3 ± 7.7 pg/ml to 229.0 ± 24.9 pg/ml, whereas in UC-MSC-conditioned media the contents of VEGF-A did not change, remaining at the level of the growth medium (Fig. 1d).

### The influence of UC-MSC-conditioned media on endothelial cell proliferation

**Fig. 2:**
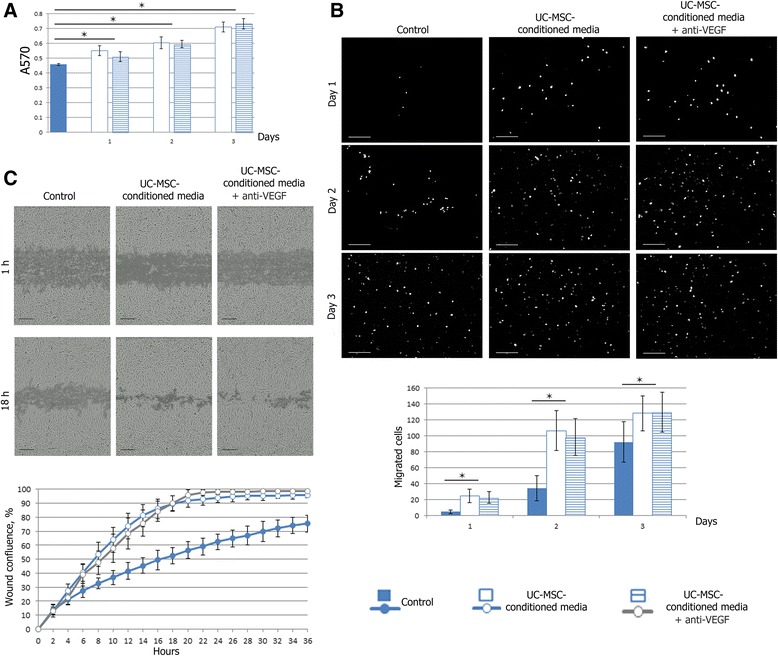
Effects of UC-MSC-conditioned media on proliferation, directed migration, and motility of EA.hy926 cells. **a** Effects of UC-MSC-conditioned media on proliferation of EA.hy926 cells was determined by MTT assay. Cells were treated with UC-MSC-conditioned media or UC-MSC-conditioned media supplemented with anti-VEGF antibody for 1, 2, or 3 days. Control cells were treated with growth media for 3 days. Values are expressed as average ± SD of three replicates. *p <0.05. **b** Migration of EA.hy926 cells to UC-MSC-released chemoattractants was measured by transwell chamber migration assay. UC-MSCs were seeded in the lower part of transwell plates, while EA.hy926 cells were placed in the upper chambers. (*Upper*) Representative images of EA.hy926 cells, which migrated to the other side of the membrane and were stained with DAPI. Scale bar 200 μm. (*Lower*) Quantification of transwell chamber migration assay. Values are expressed as average ± SD of three replicates. *p <0.05. **c** Effects of UC-MSC-conditioned media on motility of EA.hy926 cells was analyzed using wound healing assays. (*Upper*) Representative images of an in vitro scratch wound healing assay in EA.hy926 cells in the presence of UC-MSC-conditioned media or UC-MSC-conditioned media supplemented with anti-VEGF antibody, vs. growth media. Scale bar 200 μm. (*Lower*) Quantification of in vitro wound healing. Values are expressed as average ± SD of three replicates. There was a significant increase in the wound confluence exposed to UC-MSC-conditioned media or UC-MSC-conditioned media supplemented with anti-VEGF antibody compared with growth media at 8 hours after scratching. *h* hours, *UC-MSC* umbilical cord-derived mesenchymal stromal/stem cell, *VEGF* vascular endothelial growth factor

Results of the MTT assay indicated that EA.hy926 cells incubated in UC-MSC-conditioned media had a significant increase in cell viability after a 24-hour incubation when compared with cells incubated with growth media alone (*p* < 0.05). Consequently, the difference between the two media increased, and 72 hours of incubation resulted in almost 1.6-fold excess absorption in wells with UC-MSC-conditioned media compared with growth media. The addition of the VEGF-neutralizing antibody to the UC-MSC-conditioned media did not significantly attenuate the EA.hy926 cell proliferation as compared with the UC-MSC-conditioned media treatment (*p* < 0.05) (Fig. 2a).

### The influence of UC-MSC-conditioned media on endothelial cell migration

Endothelial cell migration was evaluated using a transwell chamber migration assay. EA.hy926 cells migrated from the upper chamber to the lower surface of membrane through 8 μm pores when the lower chamber contained only the growth culture medium. When the lower chamber was seeded with UC-MSCs, the efficacy of EA.hy926 cell-directed migration increased significantly at all time points (*p* <0.05). The addition of the VEGF-neutralizing antibody to the lower chambers seeded with UC-MSCs did not significantly attenuate the EA.hy926 cell migration as compared with UC-MSC-conditioned media treatment (*p* <0.05) (Fig. 2b).

Additionally we used a scratch wound healing assay of tissue-culture cell monolayers to measure the influence of UC-MSC-conditioned media on endothelial cell EA.hy926 migration. UC-MSC-conditioned media stimulated the motility of endothelial cells during the wound recovery; the difference became significant at 8 hours after scratching. For example, at 18 hours after scratching, wound confluence in the control wells was 52.68 ± 5.80 % while in the wells with UC-MSC-conditioned media this index reached 89.74 ± 5.63 %, and in the wells with UC-MSC-conditioned media supplemented with the VEGF-neutralizing antibody it reached 90.04 ± 5.26 %. The dynamics of wound confluence, illustrated with representative images from the time-lapse recording, is given in Fig. 2c.

### Tube formation assay

**Fig. 3:**
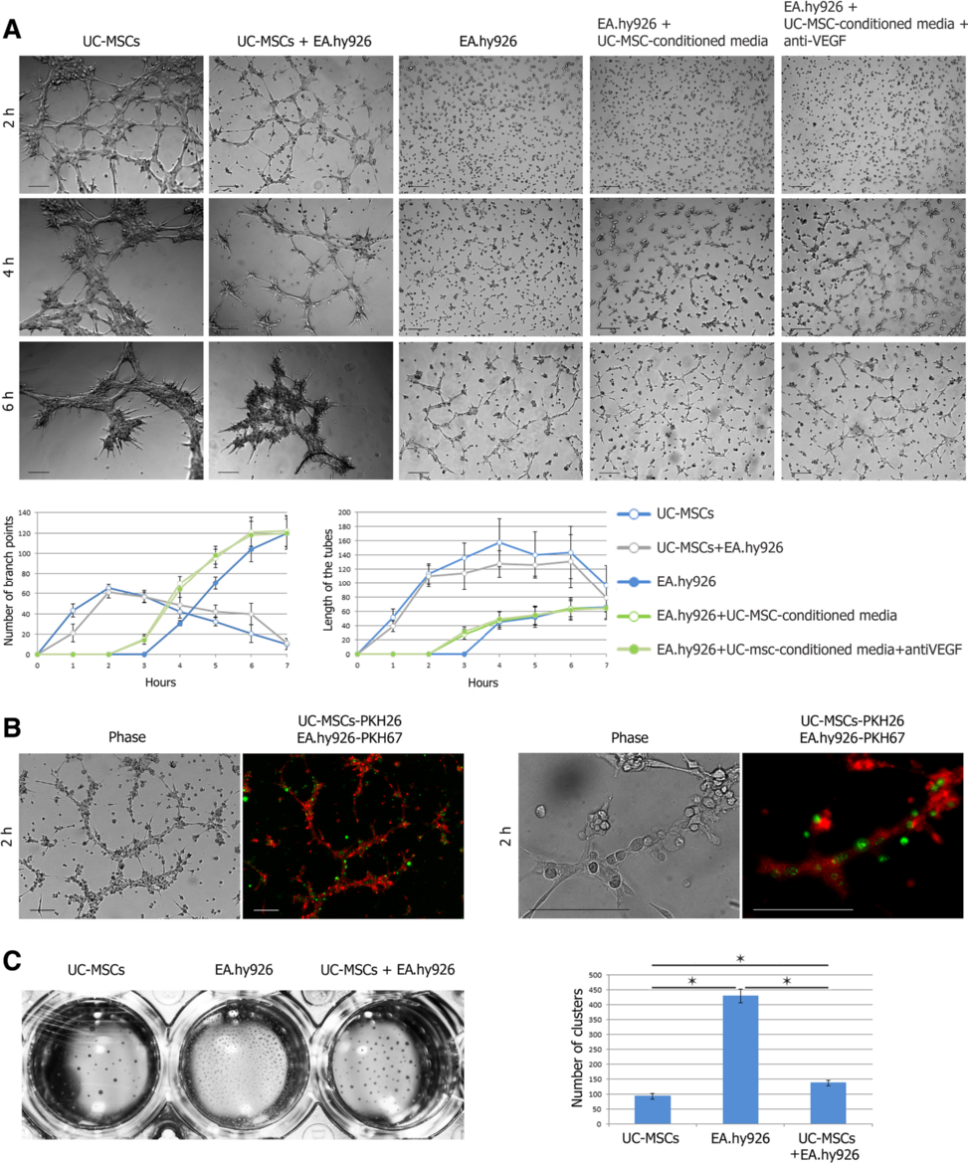
Matrigel tube formation assay. **a** UC-MSCs, or EA.hy926 cells, or 1:1 mixed UC-MSC-EA.hy926 cells in the presence of growth media, or EA.hy926 cells in the presence of UC-MSC-conditioned media or UC-MSC-conditioned media supplemented with anti-VEGF antibody (total 35 × 10^3^ cells per well) were cultured in 48-well plates coated with Matrigel. (*Right*) Representative images of morphological changes of networks. Scale bar 100 μm. (*Left*) Quantification of lengths of the tubes and numbers of branch points. Values are expressed as average ± SD. **b** PKH26-labeled UC-MSCs (*red*) became the basis of a mixed culture network, while PKH67-labeled EA.hy926 cells (*green*) were only associated with it. Scale bar 100 μm. **c** Cluster formation. (*Left*) Representative images of tight clusters formed in Matrigel by UC-MSCs, or EA.hy926 cells, or mixed UC-MSC-EA.hy926 cells 24 hours after seeding. Macrophotograph of the 48-well plate. (*Right*) Quantification of numbers of clusters. Values are expressed as average ± SD of three replicates. *p <0.05. *h* hours, *UC-MSC* umbilical cord-derived mesenchymal stromal/stem cell, *VEGF* vascular endothelial growth factor (Color figure online)

The angiogenic capability of various cell types was assessed using an in vitro capillary-like structure (tube) formation assay on basement membrane matrix. As shown in Fig. 3a, both UC-MSCs and endothelial EA.hy926 cells were able to form the networks on Matrigel, but the parameters of the networks were different. UC-MSCs and UC-MSCs mixed 1:1 with EA.hy926 cells already began sprouting 1 hour after cell seeding. These networks were unstable and began to disintegrate 3 hours later, just as EA.hy926 cells started sprouting. Additionally there was a difference between the network structures: EA.hy926 cells formed fine meshes with a greater number of branch points and shorter tubes, while the UC-MSCs and UC-MSC-EA.hy926 cell mix formed coarse meshes with fewer branch points and longer tubes. The addition of UC-MSC-conditioned media (regardless of the presence of the VEGF-neutralizing antibody) to the wells with EA.hy926 cells did not significantly alter the parameters of the networks, but contributed to their formation 1 hour earlier (Fig. 3a).

Surprisingly, we found that PKH26-labeled UC-MSCs became the basis of a mixed culture network, while PKH67-labeled EA.hy926 cells were only associated with it (Fig. 3b).

In all groups, the networks were unstable and disintegrated into tight clusters for 24 hours. These clusters were not stationary structures. In a few days, they were capable of limited movement and fusion. The movement of cell clusters stopped in about 5–7 days, and the number of clusters was different between the groups: 430.0 ± 21.2 per well for EA.hy926 cells, 137.1 ± 9.2 per well for UC-MSC-EA.hy926 cell mix, and 93.0 ± 9.2 for UC-MSCs (Fig. 3c).

**Fig. 4:**
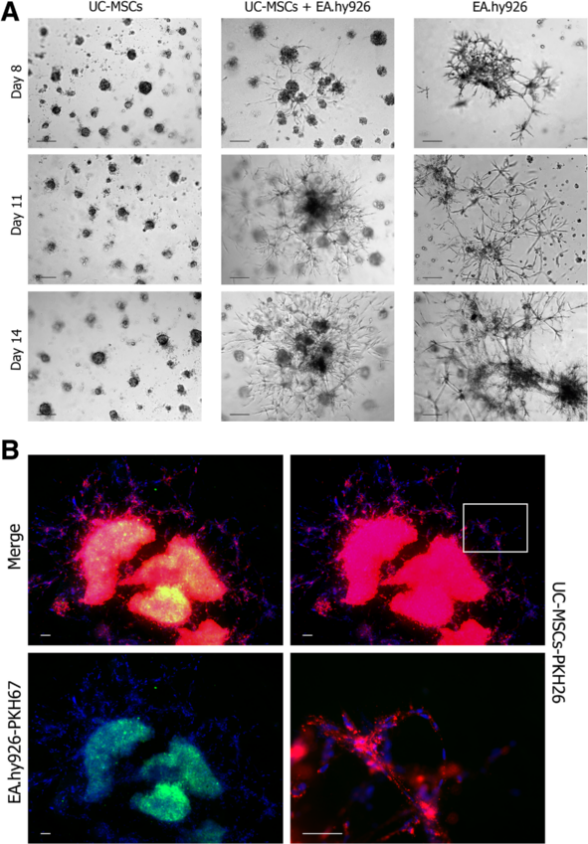
Secondary sprouting in Matrigel. **a** Clusters formed in Matrigel by the EA.hy926 cells and UC-MSC-EA.hy926 cell mix, but not by UC-MSCs alone, became centers of secondary sprouting. Gradually, the isolated sprouting centers joined into a single, very stable 3D network. Scale bar 100 μm. **b** In a mixed culture network only PKH26-labeled UC-MSCs (*red*) formed sprouts while PKH67-labeled EA.hy926 cells (*green*) stayed in the centers of the clusters. Scale bar 100 μm. *UC-MSC* umbilical cord-derived mesenchymal stromal/stem cell (Color figure online)

Further, we observed that the clusters formed by the EA.hy926 cells and UC-MSC-EA.hy926 cell mix, but not by UC-MSCs alone, became centers of secondary sprouting. Sprouting cells had a typical elongated shape. Gradually, the isolated sprouting centers joined into a single, very stable (follow-up of more than 30 days) 3D network with a plurality of branch points, often dichotomic (Fig. 4a). Moreover, in this mixed culture network only PKH26-labeled UC-MSCs formed sprouts while PKH67-labeled EA.hy926 cells stayed in the centers of the clusters (Fig. 4b).

### Endothelial differentiation of UC-MSCs in monolayer

UC-MSCs cultured in complete fresh growth medium exhibited both the shape and the wave-like arrangement of the MSCs in the monolayer; the cell growth was restricted by mutual contact inhibition, and neither of them differentiated to the CD31^+^ phenotype.

When cultured in EA.hy926-conditioned medium, the cells retained the same characteristics but grew to higher densities. When cultured in EA.hy926-conditioned medium supplemented with VEGF-A-165, they formed distinct tubular structures (three to five per 35 mm dish), assembled from several dozens of narrow stretched cells with elongated nuclei, positively stained with CD31 antibody.

**Fig. 5:**
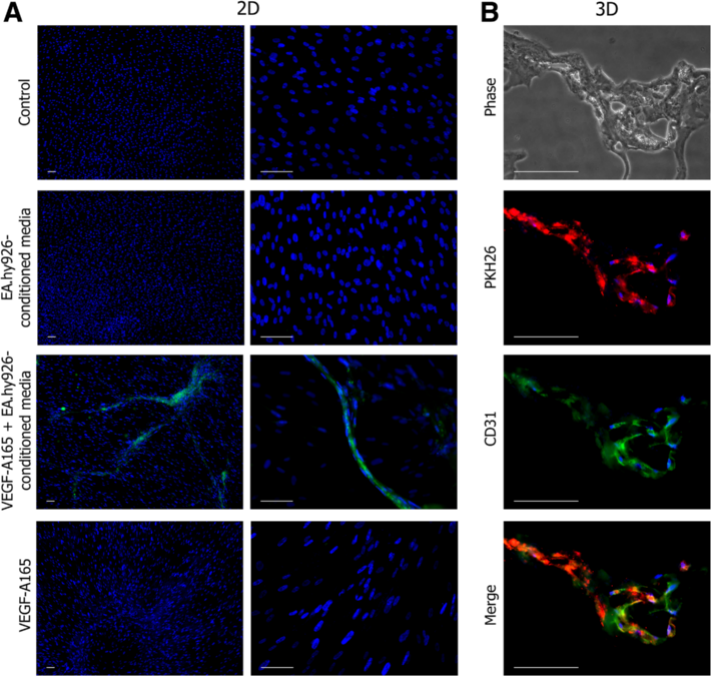
Endothelial differentiation of UC-MSCs. **a** Endothelial differentiation of UC-MSCs in monolayer. Three endothelial induction media were used for UC-MSC culture: EA.hy926-conditioned media mixed 1:1 with growth media; EA.hy926-conditioned media mixed 1:1 with growth media supplemented with VEGF-A-165 (50 ng/ml); and growth media supplemented with VEGF-A-165. Only EA.hy926-conditioned media supplemented with VEGF-A-165 led to the appearance of CD31^+^ cells in the culture. Cell nuclei were stained with DAPI. Scale bar 100 μm. **b** Endothelial differentiation of UC-MSCs in Matrigel. Cryosections of secondary sprouts promoted by PKH26-labeled UC-MSCs (*red*). Immunostaining of sections showed that, upon coculturing with EA.hy926 in Matrigel, the UC-MSCs started to express CD31 (*green*). Cell nuclei were stained with DAPI. Scale bar 100 μm. *VEGF* vascular endothelial growth factor (Color figure online)

Finally, using VEGF-A-165 as a single growth supplement (except for the serum), added to complete fresh medium, led to a mosaic loss of contact inhibition. The cells started to grow in multiple layers, but formed no tubular structures and stayed CD31^–^ (Fig. 5a).

### Endothelial differentiation of UC-MSCs in Matrigel

Immunostaining of cryosections showed that, upon coculturing with EA.hy926 in Matrigel, the UC-MSCs started to express CD31 spontaneously, without additional VEGF-A-165 supplement (Fig. 5b).

## Discussion

Weak secretion of VEGF-A by UC-MSCs derived from Wharton jelly may be related to the unusual structure of loose connective tissue forming Wharton jelly, and, in particular, the lack of blood capillaries in it. Although early events of hematopoiesis and capillary formation in this tissue are described in detail, by 7–9 weeks of development the hematopoiesis in Wharton jelly ceases, and the capillaries undergo regression [19]. It is plausible that these changes, as well as subsequent maintenance of the anti-angiogenic environment, are accompanied, or mediated, by low concentrations of soluble VEGF-A in the intercellular spaces. According to some authors, the Wharton jelly-derived UC-MSCs are able to secrete soluble forms of VEGF-A [20]; however, the majority of the reports (including this one) mention the almost complete absence of VEGF-A protein from the UC-MSC-conditioned culture medium as a specific feature reflecting VEGF-A deficiency of UC-MSC secretome. Typical levels of VEGF-A secretion reported for UC-MSCs are 10^2^ less than for adipose tissue-derived MSCs and 10^3^ less than for bone marrow-derived MSCs, despite detectable levels of transcription of the corresponding gene [12, 13]. Nevertheless, UC-MSCs can effectively accelerate migration and promote tube formation from endothelial cells in vitro*—*the effect is mediated by the UC-MSC-conditioned media; that is, by in vitro modeling of in vivo paracrine mechanisms [12].

UC-MSCs implement their pro-angiogenic potential via some VEGF-A-independent mechanism. Why should this matter? The problem is that intermediate results of clinical trials using VEGF-A-121 or VEGF-A-165 (as active exogenous proteins or in the form of genetic constructs) have been qualified as rather contradictory: the effects sometimes deviate from those expected [1, 21]. This may be explained by duality that originates from the level of VEGF-A binding to its receptor, vascular endothelial growth factor receptor (VEGFR). By acting via VEGFR2, VEGF-A increases survival and proliferation of endothelial cells, as well as recruitment of other progenitors to the site of injury, thus supporting formation and maturation of new blood vessels; in contrast, the VEGFR1-mediated action of VEGF-A is anti-angiogenic [1]. In some circumstances, excessive influx of VEGF-A (either exogenous for cells in vitro, or endogenous for ischemia models in vivo) may dysregulate intrinsic VEGFR balance of target cells and switch them to VEGFR1 expression or may upregulate soluble VEGFR1 expression that can operate as a negative feedback system, thereby undermining the entire positive effect of the treatment [22–24]. Moreover, the qualitative and quantitative balance of VEGFR1 and VEGFR2 proteins may vary in human populations, further complicating the proper choice of treatment for some cases [23]. Considering this, any VEGF-A-based pro-angiogenic therapy may prove to be more reliable and lead to more predictable results when supported by some additional VEGF-A-independent line of intervention (e.g., using UC-MSCs).

Published evidence for the stimulating influence of MSCs on endothelial cell proliferation is rather controversial because of the variety of sources and methods of obtaining the cells [2]. For example, it is shown that bone marrow-derived MSCs (including cells cultured under hypoxic conditions) have no effect on EA.hy926 cell growth [25]. In our experiments, the UC-MSC-conditioned media stimulated proliferation of EA.hy926 cells; this is consistent with results reported by Choi et al. [26] for a different endothelial line (HUVEC). The absence of VEGF-A from UC-MSC-conditioned media suggests that the endothelial cells respond to a different sort of inducer (possibly VEGF-B, the positive effect of which on EA.hy926 cell proliferation was confirmed in a recent study [27], but it is still questionable whether VEGF-B is produced by MSCs).

The transwell systems are widely used to assess chemotaxis, which plays an important role during the early stages of angiogenesis. In our setting, UC-MSCs secreted chemoattractants for EA.hy926 cells. Similar results have been reported previously for other cell lines – HUVEC, human microvascular endothelial cell line HMEC1, and mouse neural crest-derived cell line N2a, the effect of UC-MSCs being more pronounced compared with bone marrow-derived MSCs [26, 28, 29].

Similarly, the UC-MSC-conditioned medium increased the mobility of EA.hy926 endothelial cells in the monolayer scratch experiments; Bronckaers et al. [2] demonstrate the same effect for bone marrow-derived MSCs. The “scratch assay” is a common way to analyze proliferation as combined with directed migration of the cells in vitro [30]; exactly these processes play a central role in angiogenesis [31].

UC-MSCs thus secrete factors that may attract endothelial and progenitor cells, while stimulating their mobility; what kind of factors in particular could be partly deduced from the literature. Interleukin (IL)-8 is shown to induce cytoskeleton rearrangement and directed migration of EA.hy926 cells by activation of р38 mitogen-activated protein kinase signaling [32]. The rate of migration of endothelial cells in vitro is also shown to depend on hepatocyte growth factor (HGF) and monocyte chemoattractant protein-1 (MCP-1) levels in UC-MSC-conditioned media [28]. These findings are consistent with other research showing that secretion of IL-8, HGF, and MCP-1 by UC-MSCs is significantly more intensive than by MSCs derived from bone marrow or adipose tissue [13, 20].

Published data on the possibilities of endothelial differentiation of MSCs themselves are rather contradictory. There exist several protocols that differ in the composition of inducers (most widely used is VEGF-А-165 at 50 ng/ml), duration of the process of differentiation (takes from 2 to 28 days), and selection of molecular markers for the control immunostaining (CD31, von Willebrand factor (vWF), vascular endothelial cadherin (VE-cadherin), and VEGFR2 are the most common); accordingly, the final products of these protocols vary greatly [33]. At the same time, designating the differentiated MSCs as fully mature and functional endothelial cells is considered inaccurate; it is therefore more correct to define these cells as endothelial-like cells [2].

In our experiments, the UC-MSCs were capable of differentiation to the CD31^+^ phenotype under the influence of differentiation medium containing VEGF-A-165 as an essential, although insufficient, inducer. In contrast, Choi et al. [26] observe no expression of endothelial markers by UC-MSCs after treatment with complex differentiation media containing epidermal growth factor (EGF), VEGF, basic fibroblast growth factor, insulin-like growth factor-1, hydrocortisone, and some other potential inducers. In yet another study, UC-MSCs treated with media containing VEGF, EGF, and hydrocortisone started to express endothelial markers (vWF, VE-cadherin, and VEGFR2) uniformly, without any changes in cell organization or cell morphology [34]. Possibilities of endothelial differentiation of MSCs in vivo are even more questionable [2]. One of the reasons for this is the low level of VEGF-A in ischemic tissues: it is about 10^3^ times lower than in standard endothelial differentiation media (50 ng/ml) [35], and roughly corresponds to the VEGF-A level in the EA.hy926-conditioned media (Fig. 1d).

The networks formed in coculture of UC-MSCs with EA.hy926 cells on Matrigel were similar to the networks formed by pure UC-MSCs (judging by their assembly time, length of the tubes, and branching point number). The core of the mixed networks was composed of the PKH26-labeled MSCs, while the PKH67-labeled EA.hy926 cells were associated with the outer surface of this core (Fig 3a). Such an arrangement of cell types in mixed networks differs from that reported previously: other authors attribute only a minor role to MSCs [26, 36, 37]. The inconsistency probably relates to a different proportion of cell types taken for the network priming.

All tubular networks observed in our setting were unstable. Independently of whether they were formed by UC-MSCs combined with EA.hy926 cells or either of the cell types on their own, the networks underwent spontaneous disassembling in the course of 24 hours, producing tight cell clusters; this is consistent with previously published data [26, 36, 38]. These clusters, resulting from 2D network disassembly, subsequently turned into sprouting centers producing a single stable 3D network. Similar results are reported by Portalska et al. [38], who observed in vitro assembly of blood vessel-like structures from bone marrow-derived MSCs predifferentiated to an endothelial-like phenotype: the cells started to form a network with a 20-hour delay (as compared with the native undifferentiated MSCs), and this network remained stable for at least 7 days. In pure cultures of endothelial cells, as well in two of the five mixed cultures, the sprouting occurred invariably, but all five pure UC-MSC cultures showed no signs of the sprouting. This confirms the idea that individual MSC cultures, equally complying with the standards, may show morphological and functional variation [39].

Overall, the results indicate that the ability of UC-MSCs to participate in sprouting, manifested in cocultures with EA.hy926 cells on Matrigel, is a consequence of their differentiation to an endothelial-like phenotype (especially given that the signals from the local environment, either through cell–cell contact, soluble factors, or cell–matrix interactions, profoundly influence MSC endothelial differentiation [37]). We also assume that the low reproducibility of sprouting indexes between individual cultures of UC-MSCs is caused mainly by unequal susceptibility of these cultures to specific endothelial differentiation stimuli. Notably, acquisition of the CD31^+^ phenotype by UC-MSCs in long-term coculture with EA.hy926 cells on Matrigel occurred in the absence of exogenous VEGF-A. It is therefore possible that the role of VEGF-A in endothelial differentiation of MSCs is not so significant as believed previously; this inference is substantiated by the fact that MSCs do not express membrane-anchored VEGF receptors, although VEGF-A can signal through platelet-derived growth factor receptors [40].

## Conclusions

Many of the studies investigating the paracrine factors secreted from MSCs derived from various sources have reported the presence of VEGF-A and have implicated its importance in angiogenesis. In this study, we confirmed that MSCs derived from Wharton jelly of the human umbilical cord produced no detectable quantities of soluble VEGF-A (VEGF-A-121 and VEGF-A-165); despite this, culture medium conditioned by UC-MSCs effectively stimulated the proliferation, motility, and directed migration of endothelial EA.hy926 cells. These data suggest that a VEGF-A-independent paracrine mechanism is involved in the pro-angiogenic activity of UC-MSCs.

In our experiments, the UC-MSCs were capable of differentiation to an endothelial cell-like CD31^+^ phenotype under the influence of differentiation medium containing VEGF-A-165 as an essential, although insufficient, inducer. However, we found that the ability of UC-MSCs to participate in secondary sprouting, as manifested in long-term cocultures with EA.hy926 cells on Matrigel, is a consequence of their differentiation to an endothelial-like CD31^+^ phenotype in the absence of exogenous VEGF-A. We can assume that signals from the local environment, either through UC-MSC–EA.hy926 cell contact or UC-MSC–basement membrane matrix interactions, profoundly influenced UC-MSC differentiation. Endothelial differentiation as one of the proposed mechanisms of action for UC-MSC transplantation can thus also be partially VEGF-A independent.

The conclusions of this study have practical applications in the field of pro-angiogenic therapy: VEGF-A-based therapy supported by an additional VEGF-A-independent line of intervention (e.g., using UC-MSC transplantation) may have higher efficacy.

## Abbreviations

ANOVA: Analysis of variance
DAPI: 4′,6-Diamidino-2-phenylindole
DMEM: Dulbecco’s modified Eagle’s medium
DMSO: Dimethyl sulfoxide
EGF: Epidermal growth factor
FBS: Fetal bovine serum
FITC: Fluorescein isothiocyanate
HGF: Hepatocyte growth factor
HUVEC: Human umbilical vein endothelial cells
IL: Interleukin
MCP-1: Monocyte chemoattractant protein-1
MSC: Mesenchymal stromal/stem cell
MTT: 3-(4,5-Dimethylthiazol-2-yl)-2,5-diphenyltetrazolium bromide
PBS: Phosphate-buffered saline
SD: Standard deviation
UC-MSC: Umbilical cord-derived mesenchymal stromal/stem cell
VE-cadherin: Vascular endothelial cadherin
VEGF: Vascular endothelial growth factor
VEGFR: Vascular endothelial growth factor receptor
vWF: Von Willebrand factor
